# Tracking the transition of adolescents into adult HIV care: a global assessment

**DOI:** 10.7448/IAS.20.4.21878

**Published:** 2017-05-16

**Authors:** Annette H. Sohn, Rachel C. Vreeman, Ali Judd

**Affiliations:** ^a^ TREAT Asia/amfAR – The Foundation for AIDS Research, Bangkok, Thailand; ^b^ Indiana University School Medicine, Department of Pediatrics, Indianapolis, IN, USA; ^c^ MRC Clinical Trials Unit at University College London (UCL), UCL, London, UK

Emergence of the issue of adolescent-to-adult HIV care transition as a priority in the HIV response has been like an iceberg rising out of the water. In the early years of the AIDS epidemic, it did not even merit a footnote, as the world was too busy watching children die without treatment. After we had a few antiretrovirals – crushed adult-sized pills used off-label – things got better, but there were complications of delayed treatment and drug side effects that diverted our attention. We are now in an era where more of the world’s HIV-infected children and adolescents can be diagnosed and access potent combination antiretroviral therapy (ART) regimens, but our focus has more frequently been on considering how to help them maintain adherence to life-long ART to avoid treatment failure and drug resistance. In the midst of these clinical considerations, they began aging out of the traditional paediatric clinic structure. Whether or not they or their current or future providers were ready for them to move on to adult HIV care, the simple passage of time means that all of us now have to face this change.

Paediatric providers of health services for children with chronic diseases are regularly, if quietly, criticized for “holding on” to our patients for too long. With the scale-up of ART, HIV has been added to the list of conditions like congenital heart disease and cystic fibrosis where children eventually move on to adult clinics. In some high-income countries, adolescent HIV care may be extended into early adulthood [1]. In low- and middle-income countries, this is less likely to happen because of health system policies around aging out of specialist paediatric care or the use of all-ages or family-centred HIV clinics; however, adolescent development and changes in responsibility for self-care do still occur. Whatever the setting, the collective goal is for all of those diagnosed with HIV as children and adolescents to continuously remain in care, and thrive as adults. In order to know *if* this is happening, we need data on who transfers or transitions, and what their outcomes are over time. To know *how well* this is happening, we need data on the quality of transition processes and services.

For this special issue of the Journal of the International AIDS Society, we conducted a global appraisal of adolescent transition to collect these data, taking a deep dive into the issues that they face today. The articles represent a snapshot of what we think we know, and represent expert opinion on where research, implementation, and policy efforts need to be focused to document and improve the quality of adolescent and young adult HIV care in the future. The articles taken together provide a comprehensive review of the transition situation globally, and our authors have tackled many different aspects of transition, from estimates of the size of the adolescent epidemic to mental health issues, and from HIV treatment and care services to the policy response, often bringing disparate data together for the first time. The main focus is on young people who acquired HIV in childhood, but not to the exclusion of those who were behaviourally infected, for whom common as well as contrasting themes are highlighted.

## Constructing global epidemiological estimates for adolescents, and transition outcomes in Africa

Since global surveillance mechanisms have yet to track perinatally infected adolescents as a distinct group from older adults, we do not actually know how many people diagnosed as children are still living as adults. However, Slogrove et al. provide us with the most recently available UNAIDS estimates on the size of the current adolescent HIV epidemic that breaks the numbers down by younger (10–14 years) and older (15–19 years) groups [2]. Using Spectrum software and updated modelling assumptions, they explain a downward correction in the population size estimates to 1.76 million adolescents living with HIV in 2015, most of whom live in sub-Saharan Africa. Mortality data have similarly been revised, and now show that AIDS-related deaths in the 10–14-year age group peaked in 2010, and have slightly fallen since then, but have increased in the 15–19-year-old age group.

A key difficulty in studying post-transition outcomes is the inability in many national health systems to track patients as they move between facilities. Davies et al.’s analysis linking cohort data with surveillance data from the Western Cape Province of South Africa to assess adolescent outcomes after transfer provides proof of concept that such linkages are possible in the context of strong health data systems and implementation of unique patient identifiers [3]. The investigators were able to confirm that 82% of their 451 transfers were successful, and report on retention of this group across three years post-transfer, with CD4 and HIV viral load test results from the national HIV lab database. In settings with large and complex ART programs, optimizing the use of existing clinical data collection systems may be the most efficient way to monitor transition outcomes.

To complement these core data indicators, Mark et al.’s survey on the availability of adolescent-targeted HIV care services in sub-Saharan Africa highlights weaknesses and gaps in health systems that limit the delivery of HIV care to this group [4]. Their results demonstrate that there has been wide intra-regional variation in the degree to which services in sub-Saharan Africa were able to measure treatment outcomes in adolescents. A variety of services were provided to this group, but with little data on their effectiveness, and providers reported that adherence remained a major challenge when caring for adolescents.

## Transition outcomes and models for adolescent support

The next five articles provide summary reviews of transition outcome data across various regions of the world, as well as examples of specific outcome studies outside of sub-Saharan Africa. Together they offer expert perspectives about the next steps that are needed for implementers to improve the quality of care around this vulnerable stage of life. In the first review paper, Dahourou et al. summarize the approaches to transition and models of care for African youth [5]. While there are innovative and youth-friendly approaches described in the literature using dedicated teen clinics, peer educators, and social media, there were few reports on transition outcomes – particularly in Central and West Africa – which is a somewhat newer concept in the region that may be related to the family-centred care models predominantly used in the public health sector. The lack of clinic infrastructure and trained staff focused on youth support also make it difficult to scale up potential differentiated models of care.

Data were similarly lacking in much of the Caribbean, Central and South America, Eastern Europe, and the Asia-Pacific. Bailey et al. examined transition across these diverse, but largely middle-income settings [6]. They emphasized the challenges perinatally infected youth face with stigma and socio-economic disparities where HIV epidemics are more often concentrated within key populations. Although these are much smaller epidemics and cohorts compared to sub-Saharan Africa, the relative lack of external donor funding also means that it may be harder to ensure political commitment to expanding testing and clinical services, and coverage rates for prevention and treatment may be substantially lower.

Research efforts to track transition outcomes have been more successful in North America and parts of Europe. However, Tepper et al. explain that patients in their settings still frequently have to cope with socio-economic barriers to care, stigma, as well as the long-term effects of HIV disease, particularly for those who were born before effective ART was available [7]. They provide insights into the mental health and substance use problems that have been emerging in both perinatally and behaviourally infected youth, and explore the wide diversity of qualitative and quantitative outcomes that have been reported.

The next two articles report primary research findings from studies of outcomes among adolescents transitioning to adult care in the United Kingdom (UK) and Thailand, respectively. The study from the UK complements the Davies et al. paper, and links the national UK paediatric cohort to an adult HIV cohort study to study post-transition outcomes. These papers both demonstrate how in many settings the data that we need to measure transition outcomes may already exist, but not necessarily be held in the same place. In the UK cohort, Judd et al. show that CD4 was already declining among adolescents as they approached transition, and continued to fall among black males while rising in white females after transfer [8]. Although virologic suppression was stable across the time periods, it was still only ~40%, emphasizing the substantial need for better interventions to improve adherence and regimen potency in this age group.

The final paper in this group is an evaluation of the “Happy Teen Program”, which was developed to directly address transition in Thai youth. Lolekha et al. describe an intervention to improve and standardize pre-transition preparation through group and individual training sessions [9]. Developed through a multidisciplinary collaboration between the government, public health, and hospital sectors, participation in the program successfully improved knowledge around health (e.g. ART regimen, contraception), insurance coverage, and logistics around accessing HIV care. The authors provide a model of how interventions can be evaluated at the local level, and similar holistic approaches to adolescent support around transition need to be evaluated across country and income settings.

## What is so special about adolescents?

The next two papers highlight how although adolescents can be considered as their own distinct group, within that group are important differences, which in turn may require different interventions to ensure the best outcomes (no one size fits all). Lam et al. describe how perinatally and behaviourally infected adolescents have differing needs as well as some shared experiences and challenges [10]. They explain how transition data for behaviourally infected adolescents are perhaps more limited than for perinatally infected adolescents, with little data on the health outcomes of key adolescent population subgroups. Vreeman et al. [11] discuss the multitude of mental health challenges facing many adolescents with HIV, and the paucity of mental health diagnosis and support services, particularly in areas of the world where most HIV-positive adolescents reside. Their broad review highlights the diversity of challenges, from psychiatric diagnoses and behavioural disorders to self-harm and stigma. The intersection between HIV and mental health continues to be inadequately studied across all populations in resource-limited settings, despite being considered an essential component of long-term treatment success.

The issue closes with two commentaries, which both provide specific calls to action to address adolescent transition issues. Oliveras Rodriguez shares his personal perspectives as a community and policy advocate for young people living with HIV on the need to better engage youth in the adolescent HIV response and build their capacity for leadership [12]. He notes that we are collectively responsible for helping adolescents achieve equal access to care to optimize treatment outcomes. In the final paper of this issue, Mark et al. [13] outline five key strategies that will help us to move from policy to action on adolescent transition. The core of these efforts should be an adolescent-centred approach, without which we will continue to leave young people “lost in transition”, in between programs and policy agendas for children and adults.

## Conclusions

The papers in this issue are multidisciplinary and multidimensional, highlighting how inter-sectoral and multilevel responses are needed to ensure a smooth transition for adolescents moving between and through paediatric and adult HIV care and into adulthood. Many of the authors in this issue point out the lack of data on transition outcomes among young people, but when these papers are put side by side they do indicate how we are moving in the right directions. The seemingly small steps research teams are taking to disaggregate data and harmonize patient identifiers are making it possible for us to measure and evaluate treatment successes and failures, which will more reliably inform youth-related policy decisions. Clinical programme and policy efforts must also be combined with youth engagement and empowerment in order to achieve lasting success.

Adolescents living with HIV demand and deserve more attention and action to meet their care needs. If we are going to successfully control the HIV epidemic among youth, we need to recognize that while the bulk of the transition iceberg may still be below the water line, it will not remain that way for much longer.
